# Polydatin Prevents Electron Transport Chain Dysfunction and ROS Overproduction Paralleled by an Improvement in Lipid Peroxidation and Cardiolipin Levels in Iron-Overloaded Rat Liver Mitochondria

**DOI:** 10.3390/ijms252011104

**Published:** 2024-10-16

**Authors:** Itzel Reyna-Bolaños, Elsa Paola Solís-García, Manuel Alejando Vargas-Vargas, Donovan J. Peña-Montes, Alfredo Saavedra-Molina, Christian Cortés-Rojo, Elizabeth Calderón-Cortés

**Affiliations:** 1 Instituto Tecnológico Superior de Ciudad Hidalgo, Tecnológico Nacional de México, Ciudad Hidalgo 61100, Michoacán, Mexico; reynabolanositzel@gmail.com (I.R.-B.); paolasg63@gmail.com (E.P.S.-G.); 2 Instituto de Investigaciones Químico Biológicas, Universidad Michoacana de San Nicolás de Hidalgo, Morelia 58030, Michoacán, Mexico; 1371614e@umich.mx (M.A.V.-V.); 0618853j@umich.mx (D.J.P.-M.); francisco.saavedra@umich.mx (A.S.-M.); 3 Facultad de Enfermería, Universidad Michoacana de San Nicolás de Hidalgo, Morelia 58260, Michoacán, Mexico

**Keywords:** piceid, mitochondrial function, oxidative stress, free radicals, liver disease, respiratory chain, cytochromes, hydroxyl radical, Fe^2+^, iron overload

## Abstract

Increased intramitochondrial free iron is a key feature of various liver diseases, leading to oxidative stress, mitochondrial dysfunction, and liver damage. Polydatin is a polyphenol with a hepatoprotective effect, which has been attributed to its ability to enhance mitochondrial oxidative metabolism and antioxidant defenses, thereby inhibiting reactive oxygen species (ROS) dependent cellular damage processes and liver diseases. However, it has not been explored whether polydatin is able to exert its effects by protecting the phospholipid cardiolipin against damage from excess iron. Cardiolipin maintains the integrity and function of electron transport chain (ETC) complexes and keeps cytochrome *c* bound to mitochondria, avoiding uncontrolled apoptosis. Therefore, the effect of polydatin on oxidative lipid damage, ETC activity, cytochrome levels, and ROS production was explored in iron-exposed rat liver mitochondria. Fe^2+^ increased lipid peroxidation, decreased cardiolipin and cytochromes *c + c*_1_ and *aa*_3_ levels, inhibited ETC complex activities, and dramatically increased ROS production. Preincubation with polydatin prevented all these effects to a variable degree. These results suggest that the hepatoprotective mechanism of polydatin involves the attenuation of free radical production by iron, which enhances cardiolipin levels by counteracting membrane lipid peroxidation. This prevents the loss of cytochromes, improves ETC function, and decreases mitochondrial ROS production.

## 1. Introduction

The mitochondrial electron transport chain (ETC) is at the core of a myriad of functions because it establishes an electrochemical proton gradient (ΔµH^+^) across the mitochondrial inner membrane that supplies the energy necessary for these functions. Hence, disturbances in ETC function are generally catastrophic, as reflected by the severe disease phenotypes in mitochondrial diseases involving mutations in ETC proteins [1,2]. ETC complexes oxidize the reduced coenzymes NADH and FADH_2_ produced in catabolism and transfer electrons to oxygen through a series of redox centers in the complexes in exergonic reactions that drive proton pumping across the mitochondrial inner membrane for the establishment of the ΔµH^+^. In turn, the ΔµH^+^ drives ATP synthesis/hydrolysis, transport of various solutes across the mitochondrial inner membrane, transport of newly synthesized proteins into the matrix, and production of ROS, among others [3]. In addition, the dissipation of the ΔµH^+^ is a signal for the occurrence of cellular quality control processes, such as mitophagy or apoptosis [4]. Thus, electron transfer in the ETC is a primary process on which ΔµH^+^-driven processes depend.

ETC complexes are embedded in the phospholipid bilayer of the mitochondrial inner membrane, where they establish interactions with ETC proteins. Of particular importance are the interactions with the dimeric phospholipid cardiolipin, which are essential for the activity of ETC complexes [3]. In addition, cardiolipin maintains the morphology and architecture of the mitochondrial inner membrane [5]. Cardiolipin contains in its structure four acyl chains with unsaturated fatty acids, which makes it particularly prone to peroxidative damage by an excess of ROS [6]. Perturbations in the structure of cardiolipin due to its peroxidation or the decrease in its content cause a decrease in the activity of ETC by disturbing the morphology of ETC complexes and the mitochondrial inner membrane, inhibiting electron transfer and consequently causing an increase in ROS production [7]. In addition, damage to cardiolipin contributes significantly to the induction of apoptosis under pathological conditions by disrupting the binding of cytochrome *c* to the mitochondrial inner membrane and contributing to its release into the cytosol where it activates the pro-apoptotic cascade [8]. These events participate in the etiology of several pathophysiological conditions, such as Barth’s syndrome, ischemia–reperfusion injury, and neurodegenerative diseases, among others [9,10,11]. Therefore, cardiolipin protection against oxidative damage has been proposed as a possible therapeutic strategy to counteract mitochondrial dysfunction and oxidative stress involved in the development of several pathophysiological conditions [12].

On the other hand, alterations in iron homeostasis can cause iron overload in the mitochondria due to the role of this organelle in the synthesis of Fe-S centers and heme groups [13], leading to the overproduction of ROS as iron catalyzes the production of free radicals, such as superoxide anion (O_2_^•−^) or hydroxyl radical (OH^•^) [14]. Iron overload, mitochondrial oxidative stress, decreased cardiolipin content, ETC dysfunction, and excessive ROS production are features that characterize liver diseases [15,16,17]. This has led to the search for new compounds that prevent mitochondrial dysfunction and oxidative stress for the treatment of liver diseases [18]. A promising molecule with beneficial effects at the mitochondrial level is polydatin or piceid (3-hydroxy-5-[(1E)-2-(4-hydroxyphenyl)ethenyl]phenyl-β-D-glucopyranoside). Polydatin has been reported to have a hepatoprotective effect in rodents with fructose-induced metabolic syndrome [19], and in zebrafish and mice with alcohol-induced damage [20,21]. These effects were associated with an improvement in hepatic lipid metabolism, which is consistent with the increased lipid and glucose utilization induced by polydatin treatment in liver cell lines induced to steatosis with palmitic acid [22].

The beneficial effects of polydatin have been associated with its ability to increase the activity of several antioxidant enzymes, including superoxide dismutase (SOD), catalase, and glutathione peroxidase [23]. Other proposed targets for polydatin include improving mitochondrial function in various pathological conditions by preventing mitochondrial membrane potential dissipation [24], increasing complex I activity [25], enhancing ATP levels [26], and inducing mitophagy [27] and mitochondrial biogenesis [28]. On the other hand, polydatin has antioxidant capacity in the micromolar order of concentrations by trapping OH^•^ radicals [29]. Since Fe^2+^ ion can favor the production of OH^•^ radicals and mitochondria constitute an important source of this ion, we hypothesize that mitochondria may be a direct antioxidant target of polydatin by decreasing Fe^2+^-mediated lipid peroxidation, leading to better preservation of cardiolipin and cytochrome levels, improvement in ETC function, and, consequently, decreasing ROS levels. To test this hypothesis, we mimicked iron overload by treating isolated rat liver mitochondria with Fe^2+^ in the absence and presence of polydatin and determined the levels of lipid peroxidation, cardiolipin, cytochromes, ROS, and the activities of ETC complexes.

## 2. Results

### 2.1. Polydatin Prevents Oxidative Lipid Damage Induced In Vitro by Fe^2+^ in Rat Liver Mitochondria

**Figure 1:**
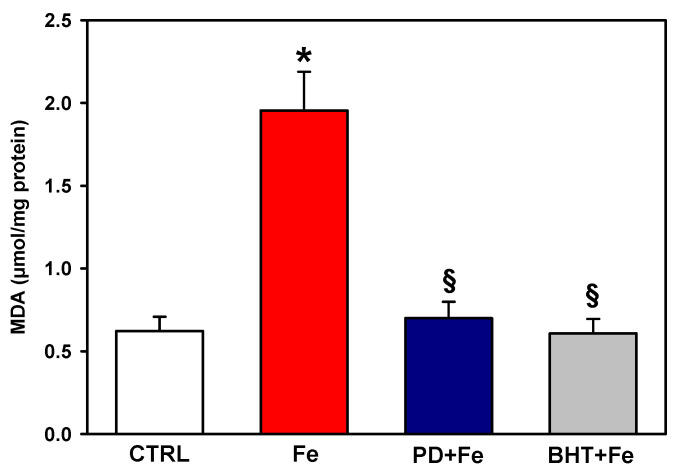
Effect of pretreatment with 100 µM polydatin (PD) or 5 µM butylated hydroxytoluene (BHT) on lipid peroxidation levels in mitochondria exposed to 50 µM ferrous iron (Fe). Results are expressed as the mean ± standard error of n ≥ 3. * *p* < 0.05 vs. CTRL and ^§^ *p* < 0.05 vs. Fe. (one-way ANOVA, Holm–Sidak post hoc test, *p* < 0.05).

Lipid peroxidation was determined in mitochondria treated with 50 µM Fe^2+^ to assess the ability of polydatin to prevent oxidative stress on mitochondrial lipids (Figure 1). Incubation with Fe^2+^ increased lipid peroxidation levels by 3.2-fold. In contrast, preincubation with 100 µM polydatin before incubation with Fe^2+^ completely prevented the increase in lipid peroxidation levels. The effect of polydatin was of a similar magnitude to that observed with 5 µM of the lipophilic antioxidant butylated hydroxytoluene (BHT), which was used as a reference.

**Figure 2:**
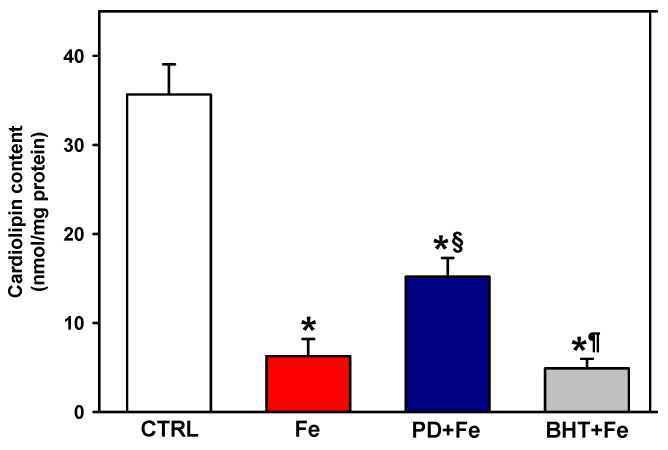
Effect of pretreatment with 100 µM polydatin (PD) or 5 µM butylated hydroxytoluene (BHT) on cardiolipin levels in mitochondria exposed to 50 µM ferrous iron (Fe). Results are expressed as the mean ± standard error of n ≥ 4. * *p* < 0.05 vs. CTRL, ^§^ *p* < 0.05 vs. Fe and ^¶^ *p* < 0.05 vs. PD + Fe (one-way ANOVA, Holm–Sidak post hoc test, *p* < 0.05).

The effect of preincubation with polydatin on mitochondrial cardiolipin levels after induction of lipid peroxidation with Fe^2+^ was determined (Figure 2). Cardiolipin levels dropped dramatically with 50 µM Fe^2+^. Pretreatment with polydatin increased cardiolipin levels 2.4-fold compared to the levels observed with Fe^2+^, although the cardiolipin concentration was 57% lower than the control. In contrast to polydatin, the antioxidant BHT was not able to prevent the Fe^2+^-induced decrease in cardiolipin levels. The results in Figure 1 and Figure 2 suggest that polydatin effectively prevents Fe^2+^-induced lipid peroxidation with a partial protective effect against cardiolipin loss.

### 2.2. Polydatin Preserves Cytochrome Levels in Rat Liver Mitochondria Exposed to Fe^+2^

**Figure 3:**
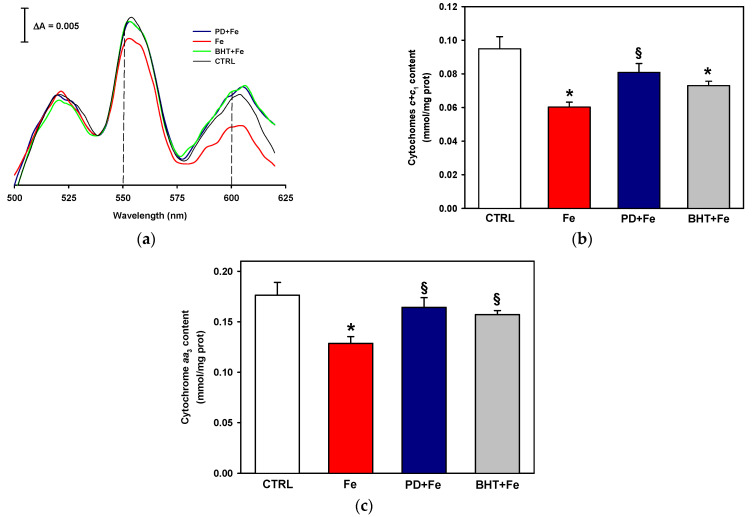
Effect of pretreatment with 100 µM polydatin (PD) or 5 µM butylated hydroxytoluene (BHT) on cytochrome levels in mitochondria exposed to 50 µM ferrous iron (Fe): (**a**) representative cytochrome differential absorption spectra. Dotted lines indicate the absorption maximum for cytochromes *c* + *c*_1_ (550 nm) and cytochromes *aa*_3_ (600 nm); (**b**) quantification of cytochrome *c + c*_1_ levels; (**c**): quantification of cytochrome *aa*_3_ levels. Results are expressed in (**b**,**c**) as the mean ± standard error of n = 4. * *p* < 0.05 vs. CTRL and ^§^ *p* < 0.05 vs. Fe (one-way ANOVA, Holm–Sidak post hoc test, *p* < 0.05).

Figure 3a shows the oxidized minus reduced spectra of cytochromes from rat liver mitochondria with or without exposure to 50 μM Fe^2+^. At 550 nm, an absorption peak maximum is observed, corresponding to the heme groups of cytochrome *c* and the cytochrome *c*_1_ of the *bc*_1_ complex of the ETC in the control (i.e., cytochromes *c* + *c*_1_, gray line). At 600 nm, an absorption peak maximum, corresponding to cytochrome *aa*_3_ of the ETC complex IV, can be observed. A drastic decrease in absorption intensity at 550 nm and 600 nm was detected in mitochondria treated with 50 μM Fe^2+^, indicative of a decrease in the content of cytochromes *c* + *c*_1_ and *aa*_3_, respectively (red line). In contrast, preincubation with 100 µM polydatin (blue line) or 5 µM BHT (green line) prior to Fe^+2^ exposure significantly attenuated the decrease in cytochrome levels, although the levels did not fully return to the control values. From these data, cytochrome concentration was quantified (Figure 3b), confirming a 40 and 30% decrease in the concentration of cytochromes *c* + *c*_1_ and aa_3_, respectively, and the prevention of the decrease in cytochrome content by pretreatment with polydatin or the lipophilic antioxidant BHT, although the effect of BHT was only statistically significant on cytochrome *aa*_3_ levels (Figure 3c). These data indicate that polydatin prevents the loss of cytochromes from the mitochondrial inner membrane, which could be related to the protective effect exerted against oxidative lipid damage observed in Figure 1 and Figure 2.

### 2.3. Polydatin Enhances the Activity of ETC Complexes of Rat Liver Mitochondria Subjected to Fe^2+^ Stress

**Figure 4:**
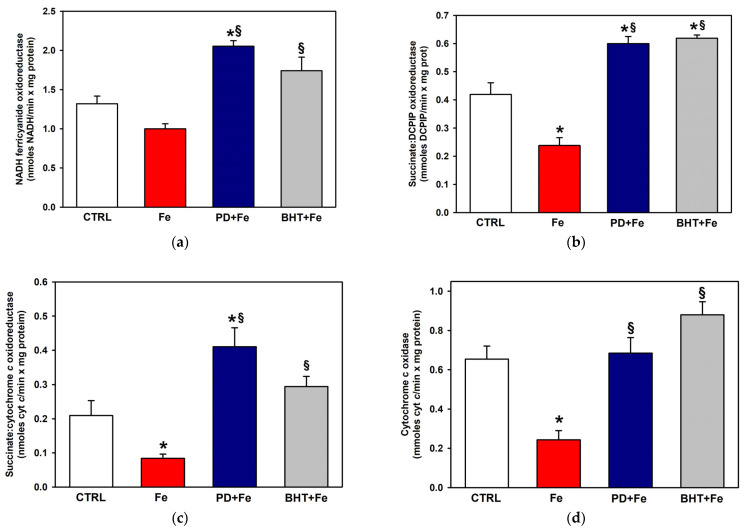
Effect of pretreatment with polydatin (PD) or butylated hydroxytoluene (BHT) on the activity of ETC complexes of mitochondria exposed to Fe^2+^ (Fe): (**a**) complex I activity (50 µM PD, 2.5 µM BHT, 25 µM Fe^2+^); (**b**) complex II activity (100 µM PD, 5 µM BHT, 50 µM Fe^2+^); (**c**) complex III activity (100 µM PD, 5 µM BHT, 50 µM Fe^2+^); (**d**) complex IV activity (200 µM PD, 5 µM BHT, 100 µM Fe^2+^). Results are expressed as mean ± standard error of n ≥ 3. * *p* < 0.05 vs. CTRL and ^§^ *p* < 0.05 vs. Fe (one-way ANOVA, Holm–Sidak post hoc test, except for (**c**), where the Student–Newman–Keuls method was used, *p* < 0.05).

The activity of ETC complexes was evaluated to determine whether polydatin could improve ETC function under Fe^2+^ stress conditions by enhancing cytochrome content and protecting against cardiolipin loss and lipid peroxidation. Complex I activity tended to decrease in a non-statistically significant manner with 25 µM Fe^2+^ (Figure 4a). Higher concentrations of Fe^2+^ were not tested due to a total inhibition of complex I that could not be recovered with either polydatin or BHT. Preincubation with BHT or polydatin enhanced the activity of complex I, which was twice as high with polydatin compared to the activity in the presence of Fe^2+^ and was even significantly higher than the control activity. Complex II activity was challenged against incubation with 50 µM Fe^2+^ (Figure 4b). This caused a decrease of 43.2% for this activity, which was entirely prevented by preincubation with BHT or polydatin. In fact, the activity with either of these antioxidants in the presence of Fe^2+^ increased statistically significantly above the control activity

Complex III underwent an inhibition of 60% upon treatment with 50 µM Fe^2+^ (Figure 3c), which was totally prevented by BHT. Polydatin statistically significantly increased this activity twofold with respect to the activity of the control. Complex IV activity decreased by 63% with 100 µM Fe^2+^ (Figure 4d). As in the rest of the complexes, preincubation with polydatin or BHT prevented inhibition of the complex by Fe^2+^.

The above results indicate that polydatin can prevent the inhibition of all ETC complexes of liver mitochondria induced by Fe^2+^ overload. This could be related not only to the protective effect of polydatin against lipid peroxidation, but also to its protective effect at the level of cytochromes and cardiolipin.

### 2.4. Polydatin Ablates ROS Overproduction in Rat Liver Mitochondria Subjected to Iron Overload

**Figure 5:**
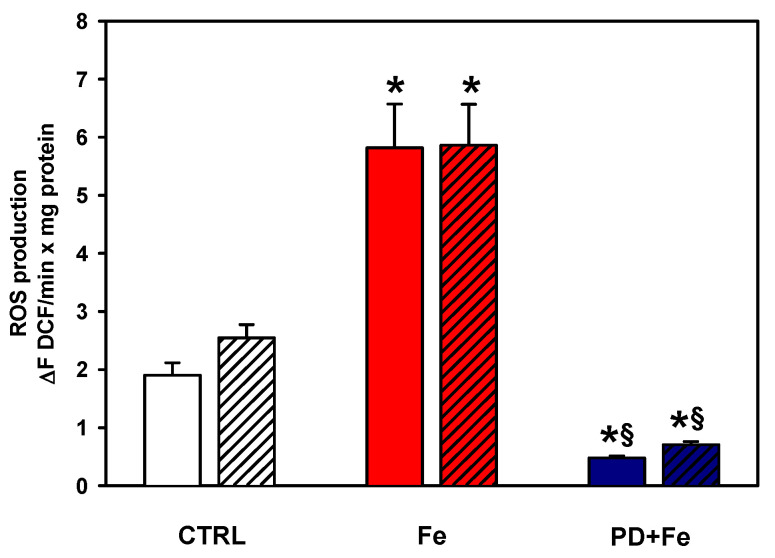
Effect of pretreatment with 50 µM polydatin (PD) on ROS levels of mitochondria exposed to 25 µM ferrous iron (Fe). Mitochondria were incubated with glutamate–malate (G + M; open bars) to stimulate ROS production and antimycin A (AA; diagonal striped bars) to stimulate maximal ROS production in the ETC. Results are expressed as the mean ± standard error of n ≥ 5. * *p* < 0.05 vs. CTRL and ^§^ *p* < 0.05 vs. Fe (one-way ANOVA, Holm–Sidak post hoc test, *p* < 0.05).

The ability of polydatin to prevent iron overload-induced ROS overproduction in rat liver mitochondria was investigated (Figure 5). ETC produces ROS when control mitochondria are energized with glutamate–malate as a complex I substrate. The addition of antimycin A, an inhibitor of complex III activity, increased ROS production, showing that the detected species are due to ETC activity. Incubation with Fe^2+^ increased ROS production almost threefold, being insensitive to the addition of antimycin A, suggesting that this increase is due to both an increase in ROS produced by ETC and ROS produced directly by Fe^2+^. Preincubation with polydatin before iron overload decreased ROS levels even below control mitochondria levels. As in control mitochondria, antimycin A increased ROS levels, but they remained below control levels. These data indicate that polydatin can mitigate ROS production not only due to the activity of ETC complexes, but also that produced by iron directly, which agrees with the protective effect of polydatin against lipid peroxidation, the decrease in cardiolipin and cytochrome levels, as well as the inhibition of ETC complexes induced by Fe^2+^.

## 3. Discussion

The results of this study suggest that the beneficial effects of polydatin under pathophysiological conditions of iron overload in liver mitochondria could be mediated by a direct antioxidant effect that decreases lipid peroxidation levels (Figure 1), partially decreases cardiolipin loss (Figure 2), and enhances cytochrome levels (Figure 3), leading to an increase in ETC activity (Figure 4) and a decrease in ROS levels (Figure 5). The addition of ferrous sulfate in acid solution in phosphate buffer, similar to the conditions used in this study, results in the production of OH^•^ radicals and the initiation of lipid peroxidation in biological membranes [30]. This agrees with the 3.2-fold increase in lipid peroxidation levels observed in Figure 1. Likewise, it has been reported that polydatin is able to scavenge OH^•^ [29], which is consistent with the total prevention in Fe^2+^-stimulated lipid peroxidation observed in Figure 1. Also, it has been reported that the antioxidant mechanism of BHT is through the neutralization of free radicals by the donation of a proton to free radicals, which prevents the extraction of a proton from a polyunsaturated fatty acid and the initiation of lipid peroxidation [31]. Therefore, the similar results obtained in lipid peroxidation with BHT and polydatin could be explained by both sharing a hydrophobic character and a similar mechanism of antioxidant action.

The depletion of cardiolipin observed with Fe^2+^ incubation (Figure 2) was expected, since this phospholipid is highly susceptible to lipid peroxidation as a result of having four highly unsaturated acyl groups [6], which increases the number of bis-allylic hydrogens susceptible to removal by OH^•^ radicals. However, the differential effects between BHT and polydatin on cardiolipin levels (Figure 2) were not expected, given that both antioxidants prevented lipid peroxidation to the same extent. It is possible that the differences between their effects lie in the lipid solubility of these two antioxidants, since BHT (calculated log P = 5.3) has a partition coefficient 3.1 times higher than polydatin (calculated log P = 1.7) [32,33]. Therefore, BHT is more likely to become stuck in the center of the lipid bilayer due to its extreme hydrophobicity, which would make it less accessible in the vicinity of the mitochondrial inner membrane where cardiolipin resides exclusively [34]. Various concerns have been raised by the use of nonyl acridine orange for the determination of cardiolipin content. For example, accumulation of this dye has been found in situ in mitochondria of *Saccharomyces cerevisiae* cells which are mutant in terms of cardiolipin synthesis [35]. On the other hand, nonyl acridine orange has been found to distribute outside the mitochondrion in cells with mitochondrial inner membrane depolarization [36]. This has discouraged the use of this dye in situ in cells and tissues [37]. In contrast, in membrane models, it has been found that nonyl acridine orange binds to cardiolipin with an affinity that is constantly two orders of magnitude higher than with phosphatidylserine and phosphatidylinosiltol, in addition to the observation that in isolated mitochondria, cardiolipin content correlates with acridine orange fluorescence [38]. Therefore, with appropriate conditions, it has been proposed that mitochondrial cardiolipin concentration can be estimated in isolated mitochondria and other membrane models [38,39].

On the other hand, while lipid peroxidation was totally prevented by polydatin (Figure 1), cardiolipin levels were partially preserved by polydatin after iron overload (Figure 2). Polydatin crosses the plasma membrane by passive diffusion and by facilitated transport via sodium-dependent glucose transporter 1 (SGLT1) [40]. In a recent study of glucose transporters localization in endomembranes, the presence of SGLT1 in mitochondrial membranes was not predicted [41]. This suggests that the accessibility of polydatin was limited by a probable process of passive diffusion across the mitochondrial outer membrane, as polydatin has an intermediate value of partition coefficient of 1.7, giving it limited accessibility to the vicinity of the mitochondrial inner membrane to trap OH^•^ radicals and prevent cardiolipin peroxidation.

Both cytochrome *c* and the heme *c*_1_ group of the cytochrome *c*_1_ subunit of ETC complex III are located in the intermembrane space. Cytochrome *c* interacts with the cardiolipin of the mitochondrial inner membrane [42], whereas heme *c*_1_ is anchored in a region of the cytochrome *c*_1_ subunit in contact with the solvent of the intermembrane space [43]. Therefore, the preservation of cytochrome *c* levels by polydatin could proceed by the protection conferred by this antioxidant to cardiolipin, decreasing its detachment from the mitochondrial inner membrane by maintaining cytochrome *c*–cardiolipin interactions. This would explain why control levels of cytochrome *c* were not reached with polydatin treatment (Figure 3), since the protection of cardiolipin by polydatin was also partial (Figure 2). Likewise, partial protection of polydatin against Fe^2+^ overload-induced damage to cytochrome *c*_1_ would be expected given its location in the mitochondrial inner membrane space and the restrictions on the accessibility of polydatin to this part of the mitochondrion noted above. Unlike the *c + c*_1_ heme groups, the *aa*_3_ heme groups of complex IV are in the core of the mitochondrial inner membrane [44]. Since lipid peroxidation was almost completely prevented with polydatin or BHT, this could explain the better preservation of heme *aa*_3_ with either of these two antioxidants than that obtained with heme *c + c*_1_, since the hydrophobic environment surrounding heme *aa*_3_ would be better preserved.

The activity of all ETC complexes was decreased by Fe^2+^ treatment (Figure 4), which was expected by virtue of the damage to the mitochondrial inner membrane induced by lipid peroxidation (Figure 1), the decrease in cardiolipin content (Figure 2), which is necessary for optimal activities of ETC complexes [45], as well as the decrease in cytochromes content (Figure 3), which are involved in electron transfer in complexes III and IV [43,44]. Since all these alterations could be prevented by preincubation with polydatin, the decrease in the activities of all ETC complexes was also totally prevented by this antioxidant. Something similar occurred with BHT, confirming that peroxidative damage to mitochondrial lipids is a central process in the inhibition of ETC by oxidative stress and that polydatin is as effective as a more lipophilic and, therefore, toxic antioxidant, such as BHT [46], in counteracting damage in a hydrophobic environment, such as the mitochondrial inner membrane.

Mitochondria produce ROS in response to substrate oxidation and these are increased by antimycin A-induced blockade of ETC in control mitochondria (Figure 5). The three-fold increase in ROS production and insensitivity to antimycin A (i.e., ROS are not increased by the addition of antimycin A), indicates that the increase in ROS was due both to defective electron transfer in ETC (Figure 4), but also by the direct production of free radicals such as the OH^•^ radical induced by the excess of added iron [14]. The decrease in ROS levels with polydatin even below control levels indicates that this antioxidant not only scavenges the radicals produced by ETC, but also the OH^•^ radical produced directly by iron, which is consistent with the ability of polydatin to scavenge OH^•^ radical as previously reported [29]. The OH^•^ radical scavenging by polydatin would be the initial step in the protective effects of polydatin against oxidative damage on mitochondrial function, as the decrease in ROS levels would decrease the peroxidation of mitochondrial membrane lipids (Figure 1), which in turn, would preserve cardiolipin levels (Figure 2). This would preserve the interactions of cytochrome *c* with cardiolipin and, in general, the integrity of the ETC complexes and their heme groups (Figure 3), resulting in improved electron flow through the ETC (Figure 4) and decreasing the ROS produced by the ETC (Figure 5).

The implications of the results of this study are related to the effect of polydatin in liver diseases involving oxidative damage, where it has been observed that polydatin can prevent apoptosis [47]. This could be related to the increase in cardiolipin levels in mitochondria induced by polydatin, since this would preserve the cytochrome c–cardiolipin interactions that keep this protein bound to the mitochondrial inner membrane, preventing its release into the cytosol and the induction of the apoptotic cascade [8]. The preservation of cytochrome levels in general and the improvement in cardiolipin levels could also be part of the mechanism that explains the protection by polydatin of the inhibition of ETC complex activity in rats with alcoholic liver disease [21], which is consistent with the result obtained in Figure 4. It should be noted that although that work and this study showed a protective effect of polydatin against the inhibition of ETC complexes by different conditions leading to oxidative stress, the effect of polydatin on cytochrome levels, cardiolipin content, and levels of lipid peroxidation in mitochondria was not determined in that work, although protection against lipid peroxidation in liver tissue was demonstrated [21].

Iron overload and lipid peroxidation are key events in the induction of cell death by ferroptosis [48]. These events have been implicated in ferroptosis liver damage in some diseases, such as hepatic ischemia–reperfusion, NAFLD, ALD, hemochromatosis, and drug-induced liver damage [49]. One of the factors that negatively regulate ferroptosis is glutathione peroxidase-4 (Gpx-4), which decreases lipid peroxidation in the mitochondria and cytosol in a glutathione-dependent manner [50]. Given that the results of our study show that polydatin is highly effective in decreasing lipid peroxidation in liver mitochondria exposed to iron overload (Figure 1), and that polydatin has been shown to decrease lipid peroxidation in the liver and to increase glutathione levels [21], this allows us to propose that one mechanism of action of polydatin against some of the liver diseases mentioned above [47] is the suppression of the initial steps of ferroptosis by substituting the function of Gpx-4 in the mitochondria and cytosol.

Regarding whether the iron concentrations used in this study are close to the free iron concentrations found in pathophysiological states affecting liver iron homeostasis, it should first be considered that the physiological concentration of free iron in liver cell mitochondria varies from between 4.8 µM to 9.2 ± 2.7 µM in one study [51], while in another study an average concentration of 12.2 ± 4.9 was found, with a range of fluctuation between individual cells from 0.3 to 19.2 µM iron [52]. On the other hand, in two studies using a rodent model of hemochromatosis, it was found that the concentration of free iron in liver mitochondria increased by 2 to 3 times, although the absolute values were not reported [53,54]. Assuming a maximum basal concentration of 19.2 µM and a 3-fold increase in hemochromatosis, this results in a concentration of 57.6 µM free iron, which is in the range used in our experiments of 25 and 50 µM Fe^2+^ in most of the experiments, except in the measurement of complex IV activity, where 100µM Fe^2+^ was used because its enzyme activity showed a low sensitivity to iron inhibition.

The main limitation of this work is that it was performed on isolated mitochondria; thus, the effect of polydatin on mitochondrial quality control processes and mitochondrial dynamics could not be evaluated. Likewise, ETC is a dynamic entity that forms supramolecular associations called respiratory supercomplexes, whose formation is highly dependent on the phospholipid cardiolipin [55]. The results of this study do not allow us to elucidate whether the improvement in ETC function by polydatin was due to enhanced formation of respiratory supercomplexes. On the other hand, the technique used to determine cytochrome levels does not allow us to distinguish between cytochrome *c* and cytochrome *c*_1_. Therefore, detection of extramitochondrial cytochrome *c* levels by Western blot is necessary.

## 4. Materials and Methods

### 4.1. Animals and Mitochondria Isolation

Male Wistar rats weighing approximately 300 g were used, which were bred in the biotherium of the Instituto de Investigaciones Químico-Biológicas, Universidad Michoacana de San Nicolás de Hidalgo (IIQB-UMSNH). The animals were sacrificed by decapitation in accordance with Official Mexican Standard-NOM-062-ZOO-1999 [56], Technical specifications for the production, care, and use of laboratory animals. This procedure was also approved by the Biosafety and Bioethics Committee of IIQB-UMSNH.

Once the liver was dissected, the mitochondria were isolated by differential centrifugation. Briefly, the liver was cut into small pieces and homogenized in glass/teflon Potter Elvehjem homogenizer filled with medium 1 at 4 °C (220 mM mannitol, 70 mM sucrose, 2 mM MOPS, 1 mM EGTA, pH 7.4). The homogenate was centrifuged at 2500 rpm and the supernatant was recovered and centrifuged at 7500 rpm. The resulting pellet was resuspended in medium 2 (220 mM mannitol, 70 mM sucrose, 2 mM MOPS at pH 7.4), then 80 µL of 0.2% albumin was added and centrifuged at 9000 rpm. Finally, the pellet was re-suspended in 500 µL of medium 2. All centrifugations were performed for 10 min at 4 °C in a Sorvall RC6+ centrifuge. Mitochondrial protein concentration was determined by the Biuret method.

### 4.2. Treatment of Mitochondria with Iron and Antioxidants

Mitochondria were resuspended in 50 mM KH_2_PO_4_ medium (pH 6.8) at the concentration indicated for each methodology. Mitochondria were incubated for 15 min with polydatin (catalog #15721, Merck KGaA, Darmstadt, Germany) or BHT (catalog # 34750, Merck KGaA, Darmstadt, Germany) at the concentrations indicated in the captions of each figure. Subsequently, FeSO_4_ was added at the concentration indicated at the caption of each figure and incubated for 30 min. The incubations were performed on ice with constant agitation. At the end of each incubation, an excess of EDTA was added to chelate the remaining iron in the medium. The FeSO_4_ stock was prepared prior to the start of each experiment in deionized water acidified with 2 drops of H_2_SO_4_ to avoid oxidation of the Fe^2+^ ion to Fe^3+^ ion. For the control samples, the incubations were performed in the same way, except that the addition of the antioxidants and Fe_2_SO_4_ was omitted. For the iron-only treatments, incubations were performed in the same manner, except that the addition of the antioxidants was omitted.

The iron concentrations for each experiment were chosen based on a previous study by our research group, where a concentration of 50 µM FeSO_4_ caused approximately half of the maximal effect on lipid peroxidation levels in rat liver mitochondria [57]. However, this concentration of iron could not be used for the measurement of complex I activity, since we observed that 50 µM Fe^2+^ fully inhibits the activity of this complex and could not be rescued by preincubation with antioxidants; thus, a concentration of 25 µM iron was selected, which was also used for the measurement of ROS levels, since larger concentrations did not further increase ROS levels. On the other hand, complex IV was found to be insensitive to inhibition by 50 µM Fe^2+^; therefore, a concentration of 100 µM Fe^2+^ was used. Also, in preliminary experiments, it was observed that a 2:1 ratio of polydatin–iron completely inhibited lipid peroxidation; hence, a 2:1 ratio of polydatin–iron was set for all experiments.

### 4.3. Lipid Peroxidation Assay

Lipid peroxidation levels were determined by the method reported by Eldelmeier et al. [58]. A 10 mM solution of N-methyl-2-phenylindole in acetonitrile and methanol (3:1; *v*/*v*) was prepared. To each tube containing 0.5 mg/mL of mitochondrial protein, freshly treated as described in the previous section, 1.3 mL of N-methyl-2-phenylindole solution and 300 µL of methanesulfonic acid were added. A 0.01 M FeCl_3_ solution was prepared and 20 µL was added to each tube and mixed on a vortex shaker. The tubes were incubated in a water bath at 45 °C for 30 min. Subsequently, 1 mL of butanol was added and incubated for 10 min. The absorbance was determined at 586 nm on a Shimadzu UV2550 spectrophotometer (Kyoto, Japan).

### 4.4. Determination of Cardiolipin Levels

Cardiolipin levels were assessed with acridine orange dye according to the protocol described by Petit et al. [38]. A total of 10 µM nonylacridine orange (catalog #A7847, Merck KGaA, Darmstadt, Germany) was added to 0.1 mg/mL of freshly incubated mitochondria as described above. The final volume of this solution was adjusted to 1 mL with 50 mM Tris/HCl buffer (pH 7.4). Samples were incubated for 10 min and centrifuged at 12,000 rpm for 10 min and the absorbance of the supernatant was determined at 495 nm. The concentration of free nonylacridine orange was calculated from a calibration curve of known concentrations of nonylacridine orange (0–150 µM). The fraction of nonylacridine orange bound to cardiolipin was calculated by subtracting the total concentration of nonylacridine orange added to the mitochondria minus the free colorant in the supernatant. The mitochondrial cardiolipin concentration was calculated, taking into account a stoichiometry of 2 moles of nonylacridine orange/mol cardiolipin.

### 4.5. Evaluation of the Activities of ETC Complexes

Subsequent to treatment of the mitochondria as described above, the mitochondria were solubilized with Triton X-100 at a final concentration of 0.01% (6 µL of a 10× Triton solution in a total volume of 600 mL) to facilitate access for substrates and inhibitors to the redox sites of the ETC complexes, as per the protocol described by Hallberg et al. [59]. The activity of Complex I was determined by assessing NADH oxidation using K_3_Fe(CN)_6_ as electron acceptor. A total of 0.3 mg/mL of mitochondria, which had undergone three previous freeze-thaw cycles, was resuspended in 1 mL of 50 mM KH_2_PO_4_ buffer and incubated with 1 μg of antimycin A and 1 mM KCN. Following a five-minute incubation period, 5 mM K_3_Fe(CN)_6_ was added, and the absorbance was measured for one minute at 340 nm using a Shimadzu UV2550 spectrophotometer. Subsequently, NADH was added, and its oxidation was monitored for a period of four minutes. The rate of NADH oxidation was calculated using the molar extinction coefficient of 6.3 mM^−1^ cm^−1^ [60].

The enzyme activity of Complex II was assessed at room temperature through the measurement of the succinate-stimulated reduction of 2,6-dichlorophenyl indophenol (DCIP). Mitochondrial permeability was achieved through the use of 0.05% Triton X-100 detergent. The reaction mixture consisted of 50 mM KH_2_PO_4_ buffer (pH 7.6), 0.3 mg/mL Triton X-100-permeabilized mitochondria, 80 µM DCIP, 1 µg antimycin A, and 0.75 mM KCN, with a final volume of 1 mL. Following a five-minute incubation period with the inhibitors, the reaction was initiated with 10 mM sodium succinate (pH 7.6). Changes in absorbance were recorded on a Shimadzu UV2550 spectrophotometer at a wavelength of 600 nm. The reduction rate of DCIP was calculated from the slope of the absorbance graph using the molar extinction coefficient of 21 mM^−1^ cm^−1^ for DCIP [60].

The reaction mixture employed to ascertain the activity of complex III comprised 50 mM KH_2_PO_4_ (pH 7.6), 0.1 mg/mL permeabilized mitochondria, 1.5 mg oxidized cytochrome *c*, and 0.75 mM KCN, in a final volume of 1 mL. Following a five-minute incubation period, the reaction was initiated by the addition of 10 mM sodium succinate (pH 7.6). The reaction was terminated by the addition of 1 µg of antimycin A. The absorbance changes were recorded on a Shimadzu UV2550 spectrophotometer at 550 nm. The rate of cytochrome *c* reduction was calculated from the slopes of the absorbance plots using the molar extinction coefficient of 19.1 mM^−1^ cm^−1^ for cytochrome *c* [60]. The reduction of antimycin A-sensitive cytochrome *c* was calculated by subtracting the activity in the presence of antimycin A and succinate from the activity stimulated with succinate alone.

To measure the enzymatic activity of complex IV, the reaction mixture consisted of 50 mM KH_2_PO_4_ (pH 7.6), 0.1 mg/mL permeabilized mitochondria, and 1 µg antimycin A, with a final volume of 1 mL. Following a five-minute incubation period, the reaction was initiated by adding 250 µg of cytochrome *c*, which had been reduced with dithionite. The absorbance changes were recorded on a Shimadzu UV2550 spectrophotometer at 550 nm. The oxidation rate of cytochrome *c* was calculated from the slopes of the absorbance plots using the molar extinction coefficient of 19.1 mM^−1^ cm^−1^ for cytochrome *c* [60].

### 4.6. Determination of Cytochrome Levels

Cytochrome content was obtained by measuring, at room temperature, reduced minus oxidized spectra of mitochondria with a Shimadzu UV2550 spectrophotometer. A total of 5 mg/mL of mitochondrial protein previously incubated with iron and antioxidants as described above was placed in reference and sample cuvettes to record a baseline. Prior to recording the reduced spectra, 0.01 g of sodium dithionite was added as a reducing agent in the reference cuvette. The spectra were evaluated between 500 and 620 nm. The cytochrome content was determined from the molar extinction coefficients and wavelengths reported in [61].

### 4.7. Assessment of ROS Production

ROS production was determined with the fluorescent probe 2′,7′-dichlorodihydrofluorescein diacetate (H_2_DCF-DA, catalog #D6883, Merck KGaA, Darmstadt, Germany). A total of 0.5 mg/mL of mitochondrial protein previously treated with iron or antioxidants, as described above, was used. The mitochondrial protein was resuspended in a buffer containing 10 mM HEPES, 100 mM KCI, 3 mM MgCl_2_, and 3 mM KH_2_PO_4_ (pH 7.4), and incubated with the fluorescent probe for 20 min at 4 °C under constant shaking. After incubation, the suspension was placed in a quartz cuvette and the basal fluorescence was recorded. After one minute, 10 mM glutamate/malate was added as a substrate for complex I. After 10 min, 1 µg antimycin A was added for 5 min. Fluorescence was measured at 30 °C on a Shimadzu RF-5301PC spectrofluorometer (λ_ex_ 491 nm; λ_em_ 518 nm).

### 4.8. Statistical Analysis of Data

Data were expressed as the mean ± standard error of n ≥ 3. Statistically significant differences between the means of each group were analyzed with an analysis of variance (one-way ANOVA), followed by the post hoc tests mentioned in the legend of each figure (*p* < 0.05), using SigmaPlot 11.0 (Systat Software, Inc., San Jose, CA, USA).

## 5. Conclusions

Polydatin prevented iron-induced mitochondrial damage under in vitro conditions by preventing increased ROS formation, which attenuated lipid peroxidation and cardiolipin loss. The above was associated with a preservation of cytochrome levels, resulting in the improved functioning of ETC complexes. These effects could mediate the beneficial effects of polydatin in liver diseases characterized by increased iron levels, excessive ROS production, mitochondrial dysfunction, and lipid damage, including cardiolipin.

## Data Availability

The raw data supporting the conclusions of this article will be made available by the authors on request.
